# Cancer patients with oral mucositis: challenges for nursing care^1^

**DOI:** 10.1590/0104-1169.0090.2551

**Published:** 2015

**Authors:** Sarah Nilkece Mesquita Araújo, Maria Helena Barros Araújo Luz, Grazielle Roberta Freitas da Silva, Elaine Maria Leite Rangel Andrade, Lívio César Cunha Nunes, Renata Oliveira Moura

**Affiliations:** 2 Master's student, Universidade Federal do Piauí, Parnaíba, PI, Brazil. Professor, Universidade Federal do Piauí, Teresina, PI, Brazil; 3 PhD, Associate Professor, Universidade Federal do Piauí, Teresina, PI, Brazil; 4 PhD, Professor, Universidade Federal do Piauí, Teresina, PI, Brazil

**Keywords:** Stomatitis, Nursing, Medical Oncology

## Abstract

**OBJECTIVE:**

to analyze nursing care provided to cancer patients with oral mucositis based on the Nursing Process (NP).

**METHOD:**

this exploratory, descriptive, cross-sectional and quantitative study was conducted with 213 patients undergoing chemotherapy and/or radiotherapy in two cancer facilities: one philanthropic and one private service.

**RESULTS:**

the participants were mainly female, aged 45.8 years old on average, with up to 11 years of schooling and income of up to one times the minimum wage. Severe mucositis was related to chemotherapy associated with radiotherapy. Only 25.3% of the patients reported having received guidance from nurses during their treatment concerning self-care. The perceptions of patients regarding quality of care did not significantly differ between the private and public facilities. The basic human needs mainly affected were comfort, eating, and hygiene. Based on this finding, one NP was established listing the diagnoses, interventions and expected results to establish an ideal, though individualized, standard of nursing care to be provided to these patients.

**CONCLUSION:**

to understand oral mucositis is crucial to establish nursing care that includes prevention based on the implementation of an oral care plan.

## Introduction

The experience of cancer patients overlaps with nursing care since the difficulties imposed by the disease and complications accruing from the therapy, such as oral mucositis, differentiate these patients who require specialized care. 

Mucositis is a toxic inflammatory response that affects the gastrointestinal tract mucosa, a sequel of radiotherapy and/or chemotherapy or bone marrow transplantation, resulting in intense pain and impaired verbal communication and eating^(^ 1 ^)^. The occurrence of oral mucositis ranges from 40% to 76% in patients undergoing chemotherapy; 75% in individuals undergoing bone marrow transplantation; and may affect 90% of the patients undergoing radiation to the head or neck. These percentages increase when chemotherapy is associated with radiation^(^ 2 ^)^. 

Instruments to measure the degree of oral mucositis are important to determining patients' self-care deficits and plan specific care actions. The instrument most frequently used is the scale presented by the World Health Organization (WHO) in 1979. It takes into account anatomical, functional and symptomatic aspects of muscositis and classifies them in degrees of 0, I, II, III, IV, which correspond to the absence of lesions up to lesions that impede the patient's eating^ (3)^. 

In addition to using instruments, nurses must realize that care provided to cancer patients should be planned and that following the nursing process (NP) is crucial. The NP should be theoretically supported to guide data collection, the establishment of diagnoses, the planning of interventions and assessment of results^(^ 4 ^)^. Three classifications are necessarily used by the third generation of NP: Diagnoses, Results and Interventions^(^ 5 ^)^, according to specific taxonomies such as the North American Nursing Diagnosis Association (NANDA)^(^ 6 ^)^, Nursing Interventions Classification (NIC)^(^ 7 ^)^ and Nursing Outcomes Classification (NOC)^(^ 8 ^)^.

Theorists^(^ 9 ^-^ 11 ^)^ support a concept of care in the dimension of human existence as a comprehensive, interactive and associative phenomenon that is generated between caregiver and recipients of care, aiming to generate comfort, help, promotion, and reestablishment of health, and relief of human suffering. From Heidegger's perspective, caring is a key provision to deal with the world, that is, the relationship with another individual is manifested in the relationship between the being-here and the being-in-the-world, which is revealed not only by occupation, but also by pre-occupation, guided by tolerance and patience^(^ 12 ^)^.

Therefore, the relevance of this study lies in the possibility of sensitizing and challenging nurses in regard to the problem of oral mucositis and encouraging professional autonomy in establishing more effective evidence-based practices. There are few studies in the nursing field addressing this problem, so it is expected to provide parameters to support practice. Understanding the experience of these patients is essential to developing technologies and planning nursing actions intended to alleviate the conditions that arise from treatments and optimize the quality of life of this population.

Given the previous discussion, the objective was to characterize the sociodemographic and clinical profiles of cancer patients with oral mucositis and analyze the NP-based care provided to these patients.

## Method

This descriptive, cross-sectional, and quantitative study was developed in two specialized cancer care centers, one philanthropic and one private facility, located in Teresina, PI, Brazil. 

The population comprised all the patients admitted with an oral mucositis diagnosis or who developed the complication during data collection, cared for either by the outpatient clinic (chemotherapy and/or radiotherapy), or who were hospitalized. The accidental probability sample was composed of 213 patients, who expressed their consent to participate in the study by signing free and informed consent forms. 

Data were collected between August 2011 and January 2012, in three stages. The first stage included the application of the form addressing the patients' sociodemographic and clinical aspects and the situation of the nursing care provided. In regard to this last aspect, we asked about the presence or absence of nursing interventions implemented among the patients, which was important to detecting how the patients recognize the role of nurses or caregivers and verify whether there were failures in care delivery. The second stage of data collection was complementing data collected to that point with data from the patients' medical records. In this stage, we verified which phases of NP had been implemented and the nature of the interventions performed for patients with oral mucositis. The third stage included physical assessment of oral mucositis by applying the WHO Oral Mucositis Grading Scale^(^ 1 ^-^ 3 ^)^. 

For this study, we stratified the severity of mucositis into two stages, mild and severe. Mild stages included 0, I, and II, in which the patient is able to maintain his/her routine diet despite mucosal erosion. The severe stage comprised degrees III and IV; in that stage, patients require either a change in their diet or are unable to eat.

The project was approved by the Institutional Review Boards at the care centers involved in the study and at the Federal University of Piauí, Brazil (CAAE: 0147.0.045.000-11).

After coding and developing the data dictionary, information collected was validated by double-entering data in Microsoft Excel spreadsheets. Afterwards, data were exported and analyzed in the Statistical Package for the Social Sciences (SPSS), version 17.0, and absolute and relative frequencies, as well as central tendencies and dispersion measures, were computed.

## Results

In regard to the sociodemographic profile of the 213 (100%) cancer patients with mucositis: 65.3% were women; 54% were between 19 and 59 years old; the average age was 45.8 years. Most (72.3%) had 11 years of schooling, while 37.1% had an income of up to one times the minimum wage and 27.2% had no income (Table 1).

**Table 1 -:** Sociodemographic profile of patients with oral mucositis (n=213). Teresina, PI, Brazil, 2012

Variables		N	%
Sex			
	Female	139	65.3
	Male	74	34.7
Age range*			
	Up to 18 years old	32	15.0
	19 to 59 years old	115	54.0
	60 years old or older	66	31.0
Schooling			
	Up to 11 years	154	72.3
	More than 11 years of study	59	27.7
Income^†^			
	No income	58	27.2
	Up to 1 times the minimum wage	79	37.1
	From 2 to 4 times the minimum wage	59	27.7
	More than 4 times the minimum wage	17	8.0
Total		213	100.0

* Age range: Mean (xx): 45.8; Median (Md):50; Mode (Mo):65; Standard deviation:21.34; Min-Max:1-89. Confidence interval (CI): 95%;

† Minimum wage/mo: R$ 622.00 (US$ 277.21), year: 2012, Brazil.

**Figure 1 -:**
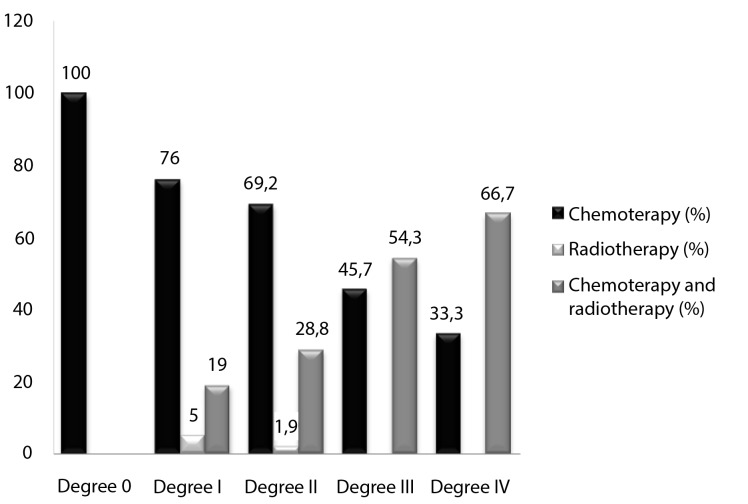
Distribution of degrees of oral mucositis according to cancer treatment proposed. Teresina, PI, Brazil, 2012 (n=211 because two patients were not undergoing cancer treatment at the time, though they were experiencing the condition due to prior treatment).

All the patients with mucositis degree 0 were undergoing chemotherapy only; 76% of those whose mucositis was classified as degree I were also undergoing chemotherapy, as well as 69.2% of those whose lesions were classified as degree II. Patients presenting more severe mucositis, degrees III or IV, were simultaneously exposed to chemotherapy and radiotherapy; 54.3% of those with lesions of degree III and 66.7% with degree IV (Figure 1).

Of the total number of patients, 74.7% reported nurses provided no treatment/orientation, while 25.3% reported orientation was provided at some point during the treatment. When the sample was segmented by degree of severity, only 27.2% of the patients with severe mucositis reported having received specific orientation from nurses (Table 2).

**Table 2 -:** Oncological nursing care according to the severity of oral mucositis (n=213). Teresina, PI, Brazil, 2012

Severity of Mucositis	Nursing care		
	Yes	No	Total
	n (%)	n (%)	n (%)
Mild*	42 (24.8)	27 (75.2)	169 (79.3)
Severe^†^	12 (27.2)	32 (72.8)	44 (20.7)
Total	54 (25.3)	59 (74.7)	213 (100.0)

* Mucositis degrees 0, I and II †Mucositis degrees III and IV

The search of the patients' records showed that only two stages of the NP were performed in both facilities: history-taking and nursing interventions. The step of history-taking was represented in the services by the nursing consultation in which a restricted anamnesis, directed only to the current pathology, and a physical assessment, addressing only vital signs and anthropometric measures, were each performed. Additionally, this consultation was not performed with all the patients due to the large demand. In regard to the interventions, the only nursing care provided was chemotherapy administration. The medical records did not present interventions concerning prevention or treatment of oral mucositis.

Only 26.2% of the patients cared for by the public facility and 24.1% from the private facility reported nurses provided specific care (Table 3).

**Table 3 -:** Oncological nursing care according to facility (n=213). Teresina, PI, Brazil, 2012

Type of facility	Nursing Care		
	Yes	No	Total
	n(%)	n(%)	n(%)
Public	33(26.2)	93(73.8)	126(59.1)
Private	21(24.1)	66(75.9)	87(40.9)
Total	54(25.3)	159(74.7)	213(100.0)

## Discussion

### Sociodemographic and clinical characterization of patients with oral mucositis

Women were most frequently affected by oral mucositis. The statistics provided by the National Institute of Cancer (INCA) corroborate this finding, as it reports an incidence of 3,330 new cases of cancer among women against 2,830 cases among men in 2012 in Piauí, Brazil. The pattern repeats in the Northeast and in Brazil in general^(^ 13 ^)^. Because women are more likely to have cancer, they are more susceptible to the adverse effects of its treatment, such as oral mucositis. 

Analyzing the most affected age ranges, we observed a predominance of women between 19 and 59 years old, coinciding with adulthood. This fact has epidemiological and social importance when we verify partial or total loss, depending on the clinical condition, of the individuals' productive potential, while at an economically active age. One study^(^ 14 ^)^ that applied the quality of life scale to cancer patients concluded that these individuals experienced decreased self-esteem, especially when they lose their role as providers and have to discontinue their work activities, seen as a genuine way to give vent to pulsions.

Most patients had attended up to 11 years of school; i.e., they had incomplete high school at most, and also had low incomes, not more than one times the minimum wage. Excluding the percentage of the sample composed of children, who were a minority, this information reveals failure in important determinants of heath, like education and income. Patients with higher levels of education and economic status deal better with the diagnosis of cancer and follow medical and nursing instructions and prescriptions more rigorously, such as for oral hygiene among those with oral mucositis^(^ 15 ^)^. Additionally, it is expected that these patients have a diagnosis in the earlier stages of the disease, avoiding the occurrence of invasive carcinomas or advanced staging of oral mucositis. Low-income individuals tend to have poor oral health conditions and nutritional deficiencies^(^ 16 ^)^. The use of educational techniques in health that adopt language appropriate to a less affluent population is essential to leading these individuals to become active subjects in the process of cure. Patient education is crucial, as well as the active participation of patients in treatment. 

In regard to the stratification of oral mucositis into degrees, note that patients with milder types of mucositis were treated only with chemotherapy, while chemotherapy combined with radiation was more frequent among those experiencing severe mucositis. Chemotherapy by itself already causes severe toxicity in the gastrointestinal mucosa and, therefore, it is the treatment that is the most determinantal of lesions. This harmful effect is strengthened when chemotherapy is associated with radiotherapy. The results are corroborated by one study^(^ 2 ^)^ that shows the occurrence of oral mucositis ranging from 40% to 76% in patients undergoing chemotherapy, but reaches 90% when it is associated with radiotherapy. 

### Nursing Process targeted to patients with oral mucositis

Ideally, whenever providing care to a cancer patient with oral mucositis, nurses should identify the level of severity and establish a classification of risk, giving priority to preventive measures for oral mucositis. Data presented in Tables 2 and 3 show that, regardless of the severity of the condition or the type of facility, the way nurses express themselves as mediating or intervening professionals, is still insufficient. This insufficiency is a fact that causes concern when we consider that the weak performance of nurses prevents the NP in oncology, which contrary to the paradigm of systematization, impedes the delimitation of indicators and assessment of results guiding improvement of care delivery. 

One ambispective study assessing oral conditions and the prevalence of oral sequelae accruing from the oncological treatment in adult patients cared for by the University Hospital of Brasilia, Brazil, detected a high frequency of oral mucositis and xerostomia and acknowledges the importance of an oral care program to prevent and treat such conditions^(^ 17 ^)^. One study conducted with the caregivers of children with cancer revealed that only 21.4% received orientation regarding care from the nursing staff^ (18)^. The authors also observed that 78.9% of the staff was not familiar with the self-care protocol concerning oral health that should be used with inpatients, reinforcing the view that the care provided by nursing technicians is fragmented as they lack specific knowledge, are insecure or are not committed to providing integral care to patients. The same was not observed in regard to nurses.

The NP should be supported based on a methodological framework and scientific model to systematize nurses' actions in order to achieve goals and results. The analysis of efficacy, effectiveness, and efficiency of care is essential for decision-making^(^ 5 ^)^. Acknowledging the importance of nurses as key players in the implementation and systematization of care, one should determine how feasible the competencies, abilities and attitudes of nurses are toward cancer patients to establish the NP. For that, it is crucial that nurses seek support in the profession's coded literature, the NANDA^(^ 6 ^)^, NIC^(^ 7 ^)^ or NOC^(^ 8 ^)^, to establish diagnoses and procedures to achieve a result that culminates in the patients' wellbeing^(^ 5 ^)^. 

To establish the NP to be implemented among patients with oral mucositis, it is essential to take into account the individual's wholeness. Continuous assessment and monitoring is paramount for efficacious management and detection of oral mucositis nursing diagnoses in order to define specific interventions that incorporate the basic principles of wound treatment. Seeking current knowledge regarding the evidence-based and time-related aspects of clinical manifestations and standardized assessments are also important for improving patients' outcomes^(^ 19 ^)^. Hence, follow-through for all the NP stages should be observed and fully implemented. History-taking of cancer patients should not only address the current disease, cancer, but also consider the individual as a whole. It should be composed of anamneses and general and specific physical assessments, taking into account the multidimensional nature of the care recipient and detecting human needs that may have been affected in order to establish nursing diagnoses^(^ 11 ^)^.

Under the domain Security/Protection, class Physical Lesion, the NANDA lists the nursing diagnosis "Impaired Oral Mucous Membrane" and related factors include radiation to head or neck and the use of anticancer agents^(^ 6 ^)^. Considering that the expected result, according to the NOC, is issues with tissue integrity of skin and mucosa and oral hygiene, both under the domain Physiological Health, class Tissue Integrity, nursing actions should be systematized to enable successful care delivery^(^ 8 ^)^. 

Patients with oral mucositis experience discomfort, pain, difficulty or inability to swallow and talk, and are susceptible to secondary infections due to deficient oral hygiene^(^ 20 ^)^. Hence, many basic human needs are affected, such as comfort, eating and hygiene.

Lack of comfort is associated with the pain the condition causes. The diagnosis of this need, according to the NANDA, is under the domain Comfort, class Physical Comfort, while Acute Pain is related to the effects of cancer treatment^(^ 6 ^)^. According to the NIC^(^ 7 ^)^, the intervention, listed under the domain Basic Physiological, class Physical Comfort Promotion, is Pain Control and possible activities include:

- To inform the patient of the cause of pain and how long it is expected to last, to minimize anxiety;
- To value the patient's pain complaints;
- Provide ideal pain relief prescribing analgesics;
- Assess the efficacy of analgesics after administration:
- Encourage diversion methods during acute pain;
- Orient patients regarding non-invasive techniques to relieve pain, such as cryotherapy;
- Apply pain assessment scales;
- Observe non-verbal signs of pain and discomfort;
- Ask a specialized physician or nurse about oral solutions to relieve pain.

Pain caused by oral mucositis is considered one of main problems associated with the cancer treatment and the use of one-dimensional and multidimensional instruments such as visual analogue scales, numerical or face scales, is needed to better assess the symptom. Experimental studies addressing new analgesic approaches are also helpful since pain is the symptom that most strongly limits quality of life^(^ 21 ^)^. Additionally, pain heavily interferes in the quality of sleep of oncological patients, especially those with head or neck cancer^(^ 22 ^)^. 

With the implementation of these interventions we expect to achieve the best prognosis for patients, which according to the NOC, is Pain Control. This outcome is under the domain Health Knowledge and Behavior, class Health Behavior^(^ 8 ^)^. 

In regard to food deficiency, this can be diagnosed using the NANDA^(^ 6 ^)^, under the domain Nutrition and class Ingestion in "Imbalanced Nutrition: less than bodily requirements" and "Impaired Swallowing," which is related to reduced oral ingestion, nausea and vomiting, secondary to radiotherapy and chemotherapy. According to the NIC^(^ 7 ^)^, under the domain Basic Physiological and class Nutrition Support is listed the intervention: Care with diet and the following activities:

- Establish the patient's daily caloric requirements together with a nutritionist, from a multidisciplinary perspective;
- Daily take anthropometric measurements to estimate weight loss;
- Enable a pleasant environment at the time of meals far away from smells or stressful situations;
- Provide instructions regarding how to prepare meals and discourage the intake of citric foods, or too spicy, sugary or salty, or fried foods;
- Eat small portions throughout the day;
- Recommend the patient quit smoking and drinking;
- Provide appropriate pain relief after meals whenever necessary;
- Encourage the intake of fluids;

One study^(23) ^addressing how oral mucositis developed among 40 patient with head and/or neck cancer for 24 months after nutritional intervention with increased protein intake reports that orienting the patient in regard to the type of food to ingest during radiotherapy reduced the manifestation of severe forms of mucositis, improving the consistency of diets and decreasing the degree of mucositis and pain.

A nutritional result is expected after implementing these activities as recommended by the NOC, which is under the domain Physiological Health, class Nutrition^(^ 8 ^)^; i.e., individuals are supposed to ingest daily nutritional requirements according to their level of activity and metabolic needs.

In regard to susceptibility to opportunistic infections accruing from inefficacious oral hygiene, the nursing diagnoses according to the NANDA are "Risk for Infection" under the domain Safety/Protection, class Infection, and "Self-care deficit: hygiene" under the domain Activity/Rest, class Self-care^(^ 6 ^)^. The first refers to the impairment of host defenses, secondary to cancer treatment, and the second refers to lack of knowledge concerning the importance of oral hygiene. According to the NIC^(^ 7 ^)^, the following interventions are listed under the domain Basic Physiological, class Self-care facilitation: Oral Health Maintenance and Infection control, and the following activities:

- Patient and workers hand washing techniques;
- Maintain isolation techniques when appropriate;
- Implement, supervise and teach correct oral hygiene care;
- Encourage the use of oxidant mouthwash solutions to combat mucous, but avoid prolonged use;
- Periodically lubricate lips and mucosae;
- Involve the family in care delivery, teaching the factors that contribute to infectious stomatitis;
- Reduce the entry of opportunistic microorganisms by implementing satisfactory oral hygiene and careful hand washing.

A systematic review conducted with 33 studies present the following Activities^(^ 24 ^)^:

- Inspect the oral cavity using instruments, such as the WHO scale, to measure the level of involvement of the oral cavity with mucositis;
- Develop individualized oral care programs according to the specificities of each patient;
- Indicate how to correctly floss;
- Encourage brushing with fluoride toothpaste after meals;
- Refer for dental assessment those patients with tooth decay, irregular restorations or who have dentures;
- Teach how to clean dentures and educate patients to abandon them when ill fitting;
- Teach how to correctly clean toothbrushes using hypochlorite;
- Recommend the use of oral antiseptics specific to each patient.

A systematic review^(^ 25 ^)^ conducted with 52 papers concluded that, regardless of the patient's age and type of oncological treatment, a plan for oral care including intense oral hygiene, determined better evidence both in regard to the prevention and treatment of oral mucositis. 

Nurses are supposed to implement and supervise oral care, always considering it a priority to provide information to the patient and help the patient the focus of the health education process, facilitating adherence and the success of nursing interventions^(^ 23 ^)^. When interventions are implemented as planned, they determine the outcome, which according to the NOC, is Self-care: oral care, which appears under the domain Functional Health, class Self-care and knowledge: infection control, listed under the domain Health Knowledge and Behavior, class Health Knowledge^(8).^

Lack of health education in nursing hinders the establishment of bonds in the multidisciplinary care chain, leaving nurses by themselves when they are required to make clinical decisions. From this perspective, the monitoring performed by nurses of deficits in basic human needs among cancer patients with mucositis is important because a precise nursing diagnosis that outlines well-directed interventions results in a positive prognosis for patients. An efficacious study of NP outlines oral care protocols that are essential components of the management of oral mucositis; when it incorporates current physiopathology knowledge to a standardized approach, such management can help reduce morbidity and improve quality of life^(^ 19 ^)^. 

## Conclusion

A lack of nursing interventions directed to cancer patients with oral mucositis was evidenced in this study, both in the public and private facilities. The administration of chemotherapy and fragmented nursing consultations were the predominant form of nursing care provided, which reveals fragile and not very considerate care delivery. As a way to ground the scientific nature and quality of care delivery, nurses should understand the profiles of susceptible patients, the main etiological factors and preventive and therapeutic measures to treat mucositis and support their practice on the NP precepts and related theories.

In conclusion, we expect that, with the interventions presented by this study, oncological patients will receive care that addresses their unique characteristics, and is able to also strengthen their souls and not only meet their physiological needs. 
